# Supernatants from lymphocytes stimulated with Bacillus Calmette-Guerin can modify the antigenicity of tumours and stimulate allogeneic T-cell responses

**DOI:** 10.1038/bjc.2011.306

**Published:** 2011-08-09

**Authors:** W M Liu, D W Fowler, A M Gravett, P Smith, A G Dalgleish

**Affiliations:** 1 Department of Oncology, Division of Clinical Sciences, St George's, University of London, 2nd floor, Jenner Wing, London SW17 0RE, UK

**Keywords:** HLA1, BCG, immunovisibility, immunotherapy, cytokines

## Abstract

**Background:**

Reduced expression of class 1 human leucocyte antigens (HLA1) is often a mechanism by which tumours evade surveillance by the host immune system. This is often associated with an immune function that is unable to mount appropriate responses against disease, which can result in a state that favours carcinogenesis.

**Methods:**

In the current study, we have explored the effects of Bacillus Calmette-Guerin (BCG) on the cytokine output of leucocytes, which is a key determinant in generating antitumour action, and have also assessed the effect of these cytokine cocktails on HLA1 expression in solid tumour cell lines.

**Results:**

BCG potently activated a broad range of leucocytes, and also enhanced the production of cytokines that were Th_1_-predominant. Supernatants from BCG-treated leucocytes significantly increased the expression of HLA1 on the surface of cancer cell lines, which correlated with increased cytolytic T-cell activity. We also showed that the increased HLA1 expression was associated with activation of intracellular signalling pathways, which was triggered by the increases in the Th_1_-cytokines interferon-*γ* and tumour necrosis factor-*α*, as counteracting their effects negated the enhancement.

**Conclusion:**

These studies reaffirm the role of BCG as a putative immunotherapy through their cytokine-modifying effects on leucocytes and their capacity to enhance tumour visibility.

It has been known for sometime that tumours possess antigens, which can be targeted by intrinsic immune processes as part of disease clearance. These tumour-associated antigens can be presented in complex with the human leucocyte antigen (HLA)-1 on their surface, which can evoke cytolytic immune responses (Yang and Yi, 2010). The principle of enhancing cell killing underscores immunotherapy for a variety of neoplasia. However, successful incorporations into clinical treatment strategies of approaches that exploit this effect have been limited (Copier *et al*, 2011). These have been due, in part, to deficiencies in the response of the immune system to tumour that have many causes; reasons have included (i) reduced immunovisibility of tumours, (ii) desensitised/anergic immune system and (iii) inappropriate immune reaction to tumours.

Specific immunity is broadly categorised as being cell-mediated or humoral, the initiation of which determines the suitability of the immune response. They can be distinguished by the types of cytokines produced by CD4+ T-cells, as cell-mediated immunity is often defined by the presence of T-cells producing Th_1_ cytokines such as interleukin (IL)2, interferon (IFN)-*γ* and tumour necrosis factor (TNF)-*α*; while humoral immunity has a profile rich in Th_2_ cytokines such as IL4, IL6 and IL10 (Abbas *et al*, 1996). These cytokines have divergent functions, and determine the nature of the response. Generally, a Th_1_-dominant response is more effective against intracellular pathogens, while a Th_2_-response is directed more towards external stimuli. It is important that the proper type of immunity is triggered to ensure an effective response. Indeed, a Th_1_-response would favour the potentiation of a CD8+ T-cell cytotoxic response against tumour (Dunn *et al*, 2002), whereas a Th_2_-response would antagonise this effect. Furthermore, a Th_2_-dominant profile is often seen in cancer patients that may favour tumour growth (Ellyard *et al*, 2007).

Treatment strategies that include systemic immune stimulation in its armoury would be powerful tools against cancer; and indeed, our group has focused on developing immunotherapies that follow such a course. Of particular interest has been our work with mycobacteria (Hrouda *et al*, 1998; Maraveyas *et al*, 1999; Nicholson *et al*, 2003). Stimulating an immune response using mycobacterium preparations such as Bacillus Calmette-Guerin (BCG) and *Mycobacterium vaccae* can enhance the cytotoxic response directed against some tumours. Furthermore, the mechanism of BCG may be mediated by Th_1_ cytokines (Orme *et al*, 1993), which evoke potent cell-mediated immune reactions against diseases. Apart from this effect, modifications to cytokine production by T-cells and other peripheral-blood mononuclear cells (PBMCs) may have other effects. For example, we have previously shown that some chemotherapies possess immune-modulatory character (Liu *et al*, 2010, 2011). This is manifest as an ability to upregulate HLA1 expression on tumours directly. As HLA1 expression on tumour cells can be induced by stimulation with type I interferons (Seliger *et al*, 2008), the effects that BCG had on cytokine production could provide a way that it could modify tumours to make them more antigenic. As cytotoxic T-cells are restricted by HLA1 and kill tumour cells expressive of them, the restoration of HLA1 expression could reinitialise immune visibility to these tumour cells and lead to an enhanced immuno-cytolytic response.

Consequently, as part of our ongoing research activities, we have worked on the hypothesis that BCG alters the cytokines produced by PBMCs that leads to a greater antigenicity of tumour cells and thus an enhanced cytotoxic immune response. Supplementary to this, we have also explored the role that signalling pathways have on this process.

## Materials and methods

### Cell culture

The human cancer cell lines A549 (lung), HCT116 (colon), MCF7 (breast) and MIAPaCa2 (pancreas) were obtained from the Cancer Research UK Cell Production Laboratories. The KM cell line was developed in house from a patient with melanoma. All cells were maintained in culture medium supplemented with 10% (v/v) foetal bovine serum (FBS), 2 mM L-glutamine and 1% penicillin/streptomycin (basal culture medium). All cell lines were incubated in a humidified atmosphere with 5% CO_2_ in air at 37 °C, and discarded when the passage number exceeded 15.

Peripheral-blood mononuclear cells were isolated from whole blood or from the residue product of leucoreduction of whole blood from pathologically healthy donors (National Blood Service, London, UK) using Histopaque-1077 (Sigma Ltd, Dorset, UK). The mononuclear fraction was harvested and red blood cell contamination removed by incubation in hypotonic ammonium chloride. Cells were washed in phosphate buffered saline (PBS) and platelet contamination removed by centrifugation at 200 **g** for 10 min, resuspended at a concentration of 1 × 10^6^ ml^−1^ in RPMI-1640 culture medium containing freshly reconstituted BCG (Danish strain 1331; Statens Serum Institut, Copenhagen, Denmark) vaccine at a concentration of 1 × 10^5^ CFU ml^−1^ (10 *μ*g ml^−1^), and incubated for 72 h in a humidified atmosphere with 5% CO_2_ in air at 37 °C. Peripheral-blood mononuclear cells were removed by centrifugation at 200 **g** for 10 min for analysis of immune cell profile. The exhausted medium was then centrifuged at ∼16 000 **g** for 10 min before storing at −80 °C, and those from BCG-treated PBMCs were designated as ‘BCG supernatants’ compared with the ‘CONT supernatant’, which were from untreated PBMCs.

### Defining immune subsets

Peripheral-blood mononuclear cells were washed twice in wash buffer (PBS containing 1% (v/v) FBS and 0.09% (v/v) NaN_3_), and stained for 30 min at 4 °C with the following antibodies: peridinin chlorophyll protein anti-CD3, phycoerythrin anti-TCR*γδ*, fluorescein isothiocyanate (FITC) anti-CD69 and allophycocyanin-conjugated anti-CD56 (all from BD Biosciences Ltd, Oxford, UK and used at 1 : 1000). Matched isotype control antibodies were used to determine background staining. Cells were washed in wash buffer and fixed in 4% paraformaldehyde (BD Biosciences) for 20 min at 4 °C. Expressions of the surface markers were analysed using a BD LSR II with dedicated proprietary software (BD Biosciences).

### Cytokine analysis of supernatants

Peripheral-blood mononuclear cells (1 × 10^6^) were plated into 96-well plates, and cultured with BCG (1 × 10^5^ CFU ml^−1^) for up to 72 h. Plates were centrifuged at 200 **g** for 10 min and supernatants removed. The concentrations of IFN-*γ*, TNF-*α*, IL10, IL5, IL4 and IL2 in these PBMC supernatants were determined by using a Th_1_/Th_2_ cytometric bead array (BD Biosciences) according to manufacturer's instructions.

### Human leucocyte antigen class I

Exponentially growing cells were reset in fresh culture medium at 2 × 10^5^ ml^−1^. Following a settling-in period of 24 h, cells were treated for 48 h with the PBMC supernatants. To study the impact of inhibiting c-Jun N-terminal kinase (JNK) on HLA1 expression, 10 *μ*M of SP600125 (Sigma) was included to the cells with and without the PBMC supernatants. Cells were harvested (1 × 10^5^) and washed twice in wash buffer, and then incubated with anti-HLA1-FITC antibody (anti-HLA:ABC 1 : 1000; BD Biosciences) for 30 min at 4 °C. Acquisition of data was performed within 1 h using a FACSCalibur (BD Biosciences). Ten thousand cells were analysed for each sample, and the mean fluorescence intensity (MFI) of HLA1 determined using the program WinMDI v2.9 (http://facs.scripps.edu/software.html).

To study the role of IFN-*γ* and TNF-*α* on modulating HLA1 expression on tumours, exponentially growing cells were treated for 24 h with PBMC supernatants in the presence or absence of anti-IFN-*γ* (AB-285-NA) and anti-TNF-*α* (AB-210-NA) (both at 10 *μ*g ml^−1^: R&D Systems, Oxford, UK). Normal goat IgG was used as the isotype control. Cells were harvested and HLA1 expression determined as described previously.

### Cytotoxicity assay

T-cells were isolated from pathologically healthy donor buffy coats (National Blood Service) using sequential negative cell selection with magnetic beads coated with anti-CD3, followed by positive cell isolation using magnetic bead coated with anti-CD8 (Miltenyi Biotec Ltd, Surrey, UK). CD8+ purities were assessed by flow cytometry, and only those preparations with purities >90% were used. Tumour cells pretreated with PBMC supernatants for 48 h were reset in culture medium at 1 × 10^4^ per well in a 96-well plate, and allowed to adhere before adding CD8+ T-cells at an effector:target ratio of 20 : 1. Maximum lysis was established by culturing cells with 1% triton-X. After a 24-h incubation period, cell-free media were removed for assessment of lactate dehydrogenase (LDH) release using a proprietary assay kit (BioVision Research Products, Mountain View, CA, USA).

Data were presented as %cytotoxicity, which was calculated from absorbance values at 590 nm using the following formula:



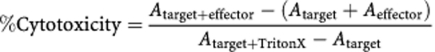



### Immunoblotting analysis

Cells were harvested; total cellular protein was solubilised in lysis buffer (New England Biolabs, Hitchin, UK) and resolved by Tris-glycine electrophoresis using a 4–12% bis–tris gradient gel. Following transfer of proteins to 0.45 *μ*m nitrocellulose membranes, blocking was performed in 5% (w/v) non-fat milk in TTBS (0.5% (v/v) Tween-20 in tris buffered saline (50 mM Tris base with 150 mM NaCl; pH 7.6)). Primary antibody probing was performed with anti-AKT, anti-phospho AKT, anti-ERK, anti-phospho ERK, anti-JNK, anti-phospho JNK, anti-p38 MAPK and anti-phospho p38 MAPK. All primary antibodies were obtained from New England Biolabs and used at a dilution of 1 : 1000, unless stated otherwise. Anti-GAPDH was used as a loading control (1 : 2000–New England Biolabs). Following three washing steps in TTBS, horseradish peroxidase-conjugated antispecies IgG1 was used as the secondary antibody (Amersham Biosciences Ltd, Little Chalfont, UK). Bands were visualised by the ECL-plus detection system (Amersham).

## Results

### BCG alters the activation status of immune cell subsets

**Figure 1:**
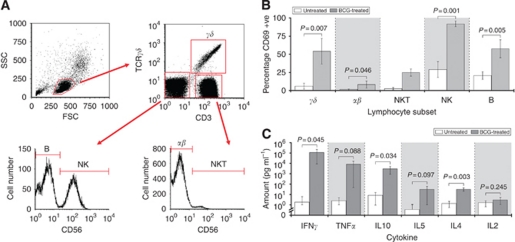
Effect of BCG on the active status of immune cells and cytokine production. Peripheral-blood mononuclear cells harvested from pathologically normal subjects were exposed to BCG for up to 72 h before assessing the percentage of CD69-expressing cells by nested flow cytometric analyses. The gating profile (**A**) allowed for identification of five immune subsets, and the percentage of CD69 positive cells assessed (**B**). The amounts of six cytokines produced by PBMCs in response to BCG were also assessed (**C**). Each data column represents the mean and s.d. of five separate individuals at the 72-h time-point, except NKT, where *n*=2 as only 2/5 donors had detectable levels of NKT cells. *P*-values from paired *t*-tests.

Peripheral-blood mononuclear cells harvested from healthy subjects were exposed to BCG for up to 72 h before assessing the activation status of each of the key immune effector subsets by flow cytometry. Activation was indicated by CD69 expression, and our gating strategy is defined in Figure 1A. Results showed that BCG was capable of increasing the percentage of CD69 expression in all the cell types studied, and that the increase was apparent as early as 6 h. Data for 72 h are shown in Figure 1B. Furthermore, the effect of BCG on CD69 expression on each of the cell types was robust and consistent in all five replicates.

### BCG stimulates cytokine production

The effect of culturing PBMCs with BCG on a number of Th_1_/Th_2_ cytokines was assessed by cytometric bead analysis, and results showed that BCG generally increased the amount of cytokines detected in the supernatants. This effect was apparent as early as 6 h, and data for the 72 h time-point is shown in Figure 1C. Results suggested a Th_1_-biased effect of BCG; specifically, although there were significant increases in the majority of the cytokines assessed, the extents to which IFN-*γ*, TNF-*α* and IL10 were elevated were greater than for IL5, IL4 and IL2. For example, at 72 h, although BCG resulted in significant increases in both IFN-*γ* and IL4 compared with untreated controls, the former was increased to a concentration of ∼115 000 pg ml^−1^, while the latter was increased to ∼32 pg ml^−1^. Furthermore, the concentrations of these two cytokines were similar in unstimulated control PBMCs (1.8 *vs* 1.4 pg ml^−1^, respectively).

### BCG supernatant increases HLA1 expression on some tumour cells

**Figure 2:**
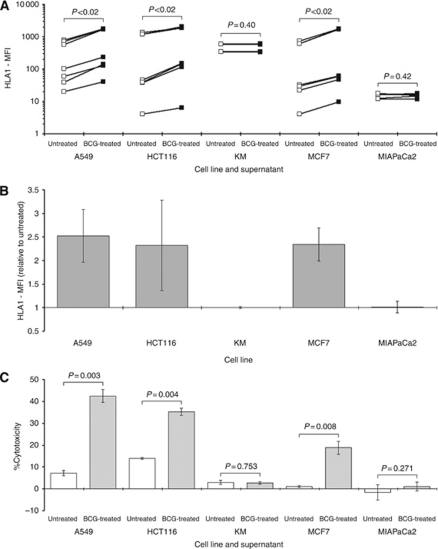
Effect of supernatants on HLA1 expression in tumour cells. Five tumour cell lines were cultured for 24 h with the supernatants derived from PBMCs treated with BCG (BCG-supernatant), before assessing HLA1 expression on the tumour cells. The basal levels of HLA1 expression expressed as MFI relative to the isotype control are presented in the text. The magnitudes of these expressions were compared with those on tumours after stimulation with supernatants from untreated PBMCs (CONT-supernatant). Data were presented as raw MFI (**A**) or relative to the respective controls (**B**). The relationship between HLA1 expression and cytotoxic effect of allogeneic CD8+ T-cells were assessed by measuring LDH activity (**C**). Effector:target ratios were at 20 : 1 and each data column represented the mean and s.d. of at least three separate experiments. *P*-values were from paired tests.

The expression of HLA1 was assessed by flow cytometry using a proprietary antibody directed against HLA–ABC and presented as MFIs. Our results confirmed that basal HLA1 expression varied in the cell lines (Liu *et al*, 2010). The order of expressions were KM <A549 <MIAPaCa2 <MCF7 <HCT116 (MFI normalised to isotype control: 0.91±0.0076; 7.5±0.61; 9.1±0.93; 34±2.7; 41±3.9, respectively). There was little effect on HLA1 expression of culturing KM and MIAPaCa2 cells with BCG supernatant. However, in A549, HCT116 and MCF7 cells culturing with BCG supernatant caused significant increases in HLA1 expressions (Figure 2A); for example, mean MFI in MCF7 cells treated with BCG supernatant was 760 *vs* 290 in CONT-supernatant-treated cells (*P*<0.02). For clarity, data were also presented relative to the CONT supernatants (Figure 2B).

### HLA1 expression is associated with cytolytic T-cell function

Allogeneic CD8+ T-cells were harvested from pathologically normal subjects and admixed with tumour cells pre-exposed to PBMC supernatants. The extent of cell death was established by assessing LDH activity in the medium by using a proprietary kit. Results indicated significant increases in the percentage of cytotoxicity following treatment with BCG supernatant only in those tumour cells with increased HLA1 expression (Figure 2C). For example, cytotoxicity in MCF7 cells was increased from a basal level of just 1.0±0.42% to 19±3.0 when BCG supernatant was used (*P*=0.008).

### BCG supernatants alter the expressions of important signalling molecules

**Figure 3:**
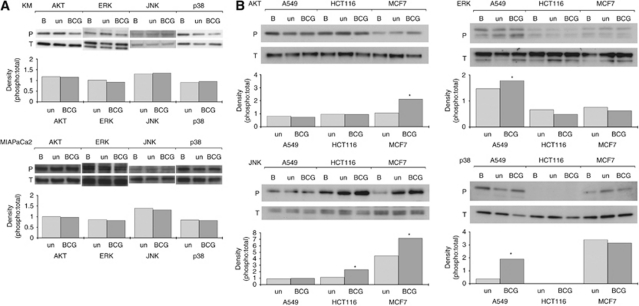
Effect of supernatants on intracellular signalling proteins. Cells were cultured with BCG supernatants for 24 h before western blotting for the proteins indicated. AKT, ERK, JNK and p38 MAPK were the proteins assessed as they represented a broad range of signalling elements indicating receptor activation and intracellular functioning. Samples were designated ‘BCG’ if treated with BCG supernatants, and expressions were compared with those from tumours cultured with CONT supernatants (‘un’) and basal expressions (‘B’: where tumours were cultured in standard medium). Both the phosphorylated (P) and total (T) levels were assessed, and densitometry were performed showing the effect of supernatants on the phospho:total ratio. Cell lines that were unaffected by BCG supernatants with regard to HLA1 expression are shown in (**A**), while those exhibiting increases in HLA1 expression following treatment in BCG supernatant are shown in (**B**). ^*^P<0.05 when compared with the untreated control as determined by paired *t*-tests.

To investigate whether the changes in HLA1 expression and/or cytotoxic responses could be associated with changes in intracellular signalling pathways, whole cell lysates obtained from the five cells cultured with supernatants were analysed by immunoblotting. A broad panel of proteins representing key protagonists in a number of signalling cascades downstream of receptor tyrosine kinases was surveyed. The intention was to employ this approach to identify a signalling protein most likely to trigger the phenotypic changes. Results highlighted differences in the cellular responses between the cells exposed to supernatants. In those cell lines where HLA1 was shown to be unaffected by BCG supernatant, there were no clear changes to the expressions of the signalling molecules studied (Figure 3A). However, in those cells where HLA1 was affected by BCG supernatants, there were changes seen (Figure 3B). Generally, there were small changes to protein expressions; however, the clearest increases were observed in JNK signalling, which happened to be common to two of the three cell lines studied (Figure 3B). Significant increases were also seen to the phospho expressions of AKT in MCF7 and p38 MAPK in A549.

### Disrupting JNK signalling affects HLA1 upregulation by BCG supernatant

**Figure 4:**
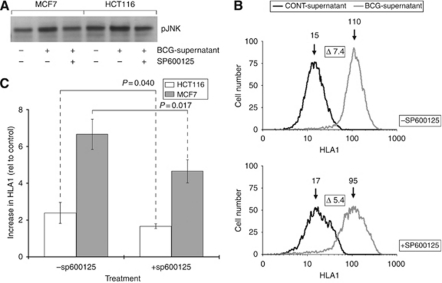
Effect of JNK inhibition on HLA1 expression. HCT116 and MCF7 cells were treated with BCG supernatant +/− the JNK inhibitor SP600125, and the effect of reduced JNK action on HLA1 expression assessed. The increase in phosphorylated JNK induced by BCG supernatant was negated by SP600125 (**A**), and the ability of BCG supernatant to enhance HLA1 expression compromised by JNK inhibition (**B** and **C**). Representative histograms from MCF7 experiments are shown (**B**), and each data column shows means and s.d.'s of a minimum of four separate experiments.

To assess the impact of JNK signalling on HLA1 expression, we cultured HCT116 and MCF7 cells with SP600125, an anthrapyrazolone inhibitor of JNK (Bennett *et al*, 2001), and assessed the extent to which HLA1 expression was altered by BCG supernatant. Early results showed that treating cells with SP600125 did not completely reverse the HLA1-enhancing effect of the BCG supernatant. However, results showed that culturing cells in the presence of SP600125 caused a significant reduction in the ability of BCG supernatant to enhance HLA1 expression in the cells (Figure 4A–C). For example, an active concentration of SP600125 reduced the increase in HLA1 expression in MCF7 cells caused by BCG supernatant from 7.4-fold to 5.4-fold (Figure 4B).

### Neutralising IFN-*γ* and TNF-*α* affects HLA1 upregulation by BCG supernatant

**Figure 5:**
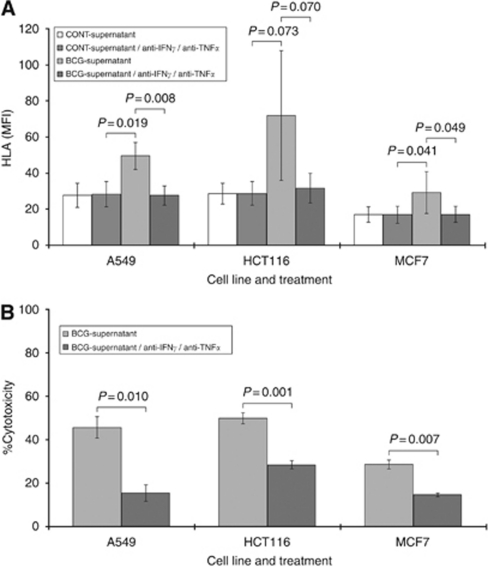
Effect of anti-IFN-*γ* and anti-TNF-*α* on HLA1 expression. A549, HCT116 and MCF7 cells were cultured with BCG supernatant in the presence or absence of neutralising antibodies against IFN-*γ* and TNF-*α*. Antagonising these cytokines caused reversal of the increases in HLA1 seen as a consequence of BCG-supernatant culture (**A**). Furthermore, culturing with the neutralising antibodies disrupted the extent of cytotoxicity seen with admixing with CD8+ T-cells (**B**). Each data column represents the mean and s.d. of at least three separate experiments.

To explore the effects of IFN-*γ* and TNF-*α* in BCG supernatants on modulating HLA1 expression on A549, HCT116 and MCF7 cells, their activities were disrupted by using antibodies against them. Results showed that these neutralising antibodies significantly reversed the HLA1-enhancing effects of the BCG supernatant in the three cell lines tested (Figure 5A). Furthermore, the enhanced cytotoxicity afforded by T-cells that was associated with increased HLA1 expression was negated by this co-culture with the neutralising antibodies (Figure 5B).

## Discussion

This study was undertaken as part of our larger remit to investigate the role of immunotherapies in an oncological setting, with particular interest in understanding the value of combining them with other modalities. These combinations could include agents that both directly and indirectly contributed to an improved action. In the current study, we specifically investigated the immunopotentiating effects of BCG, and explored their direct effects on PBMCs, as well as the effects that supernatants derived from these preparations could have on tumour cells directly. In summary, our results showed that BCG potently increased the activity of a number of immune subsets, which lead to significant potentiation of the cytokine output of these cells. Additionally, the supernatants in which they were found significantly increased HLA1 expression on a number of tumour cell lines. Increased immunovisibility was dependent upon these cytokines and associated with enhanced cytolytic T-cell activity. Moreover, the ability of tumour cells to upregulate HLA1 was partially mediated by JNK signalling.

A large number of malignant diseases are associated with suppression of cell-mediated immune responses, but humoral immunity remains unchanged or is increased (Dalgleish and O’Byrne, 2002). This presents the interesting notion that oncogenesis can sabotage cell-mediated immunity by biasing the Th_1_-/Th_2_-response towards a Th_2_-dominant immune profile that supports cancer growth (De Monte *et al*, 2011). We and others have suggested that adjusting this ‘immune rheostat’ to a Th_1_-dominant setting can restore potent cell-mediated immunity, and result in objective tumour responses (Heriot *et al*, 2000; Dalgleish and O’Byrne, 2002). These approaches could include BCG, which is known to alter the profile of cytokine output of PBMCs (Alexandroff *et al*, 1999; Luo *et al*, 2003). For that reason, in the first part of our study, we cultured freshly harvested PBMCs from pathologically normal individuals, and assayed their responses to BCG vaccine used at a clinically achievable concentration (1 × 10^5^ CFU ml^−1^). Our results showed that BCG significantly increased the percentage of cells expressing the early activation marker CD69, and that these increases were seen in all the immune subsets surveyed. Subsequent analysis of the cytokines released into the supernatants from PBMCs following BCG treatment revealed higher levels of IFN-*γ* in comparison with IL4, suggestive of a Th_1_-predominant response supportive of antitumour action. Although the specific source of these cytokines was not identified in the current study, we have previously seen significant increases in intracellular levels of IFN-*γ*, TNF-*α* and IL2 in CD3+ CD4+ cells following stimulation with BCG and other mycobacteria preparations (data not shown). Nevertheless, what was clear was a clear and significant increase in the Th_1_-cytokines IFN-*γ* and TNF-*α* that are profoundly associated with the regulation of HLA1 (Seliger *et al*, 2008), which highlights another facet of BCG that is exploitable therapeutically.

Manipulating tumour HLA1 expression as a means of potentiating the efforts of cytotoxic effectors is complex. There appears to be a fine balance in which levels are kept low to ensure evasion of CD8+ T-cells surveillance. However, loss of HLA1 opens the door to innate immune responses involving NK cells, which serve to clear cells that are devoid of or low in HLA1 (Du and Wang, 2011). Thus, restoring HLA1 expression in this way could inadvertently have the opposite effect of protecting cells from therapy by rendering tumour cells refractory to NK cell-mediated lysis (Storkus *et al*, 1991). These considerations need to be made when manipulating HLA1 expression, but it is worth noting that selective pressures on tumours results in reductions rather than increases in HLA1, which suggests that evasion of the adaptive response may be the more favourable outcome (Cabrera *et al*, 2003; Aptsiauri *et al*, 2007). These reductions can be caused by the loss of transcription and translation elements (Garrido *et al*, 1993) or epigenetic modifications that silence regulatory genes (Tomasi *et al*, 2006). Losses can also result from dysregulations of cellular signalling pathways, which are commonly altered in cancer cells, as well as from reduced responsiveness to cytokines (Rodríguez *et al*, 2007). Therefore, therapeutic interventions that can counter these oncogenic transformations and/or restore immunovisibility would be of value.

We have previously reported similar HLA1-modulating actions of some chemotherapies (Liu *et al*, 2010), and so adopted similar models to investigate the effect that the exhausted media from PBMCs cultured with BCG could have on HLA1 expression in a panel of tumour cell lines. Results showed HLA1 to be significantly increased in a number of cell lines, some of which had intrinsically low starting levels, and that these enhancements were associated with clear improvements in the cytotoxic actions of allogeneic CD8+ T-cells. Our results thus support the notion that BCG supernatants can increase HLA1 expression on tumour cells, and therefore, indirectly enhance tumour clearance. While it is already known that BCG can stimulate PBMCs to produce IFN-*γ* (Luo *et al*, 2003), and that recombinant IFN-*γ* can enhance HLA1 expression (Seliger *et al*, 2008), our data unite these two concepts by highlighting the ability of supernatants from BCG-treated PBMCs to boost HLA1 expression directly. Furthermore, the basal levels of HLA1 expressions on tumours do not directly influence their ability of being upregulated by these BCG supernatants. The tantalising possibility that other mycobacteria possess similar HLA1-boosting effects is currently being explored.

We next assessed the impact of BCG supernatants on four molecules downstream of cytokine receptors, which represented key components of intracellular signalling involved in the growth and development in cancer cells (Sebolt-Leopold and English, 2006). Our intention was to identify components that were common to the cell lines tested and not to fully deconvolute complex interplay between the cascades. Of particular interest was JNK, which was altered in two of the three cell lines studied. Antagonising JNK activity in tumour cells exposed to BCG supernatant significantly interfered with its ability to increase HLA1, which supported the important role of JNK signalling and HLA1 expression in tumour cells. Moreover, the JNK pathway has a prominent role in regulating HLA1 expression in tumour cells, whose activity is determined by IFN-*γ* signalling (Bach *et al*, 1997). In support of the observations on JNK signalling, we also explored further the roles of IFN-*γ* and TNF-*α* in increasing HLA1 expression on tumours. Our results showed that antagonising the actions of these cytokines by using neutralising antibodies against them negated the increase in HLA1 expression caused by BCG supernatant. Moreover, the enhanced cell death associated with increased HLA1 was also lost (Figure 5). This has been seen previously in the context of melanoma, where disrupting IFN-*γ* signalling can adversely affect HLA1 functionality in tumours (Rodríguez *et al*, 2007; Respa *et al*, 2011). Parenthetically, the melanoma cell line KM that was used in our study was refractory to BCG supernatant, and moreover, culturing them directly with active doses of recombinant IFN-*γ* had no effect on HLA1 expression (data not shown). This suggests differences in the mechanisms of these cytokines in modifying HLA1 that are cell line dependent. Further work to deconvolute these signalling pathways is ongoing, and will go someway in helping to identify drug partners for combination therapies, which may vary and be patient/tumour specific.

In summary, studies have suggested that the induction of an effective immune response could assist in the elimination of tumour. Approaches have included using DC vaccines to stimulate T-cell responses, and potentiating the cytolytic actions of T-cells through the use of cytokines (Copier *et al*, 2011). However, to date, successes have been limited. In spite of this, one area that has gained momentum recently is the use of conventional chemotherapy as immune stimulators. This exploits the multiple mechanisms of their action and utilises the immune-related effects of some chemotherapies that support a cell-mediated effect. For this reason, competent T-cell and NK cell functions are understandable pre-requisites to maximising the benefits of this approach. Therefore, individuals with a compromised immune system, or those possessive of one that is biased towards a Th_2_-response, may not benefit as much. For this reason, employing BCG as an adjuvant to these chemotherapies could be clinically useful for two reasons; first, by restoring the immune rheostat to an antitumour setting, and second, by restoring immune visibility of tumours via the HLA1-enhancing properties. Taken together, these data support a combination strategy, in which chemotherapy is supplemented with systemic immune stimulation, and that this approach would be a powerful tool in cancer therapy.
